# Over 20% of marine fishes shifting in the North and Barents Seas, but not in the Norwegian Sea

**DOI:** 10.7717/peerj.15801

**Published:** 2023-08-31

**Authors:** Cesc Gordó-Vilaseca, Laurene Pecuchet, Marta Coll, Henning Reiss, Alexander Jüterbock, Mark John Costello

**Affiliations:** 1 Faculty of Biosciences and Aquaculture, Nord University, Bodø, Norway; 2 The Norwegian College of Fishery Science, University of Tromsø, Tromsø, Norway; 3 Institut de Ciències del Mar (ICM-CSIC) & Ecopath International Initiative (EII), Barcelona, Spain

**Keywords:** Latitudinal shifts, Climate warming, Demersal fish, Fish communities

## Abstract

Climate warming generally induces poleward range expansions and equatorward range contractions of species’ environmental niches on a global scale. Here, we examined the direction and magnitude of species biomass centroid geographic shifts in relation to temperature and depth for 83 fish species in 9,522 standardised research trawls from the North Sea (1998–2020) to the Norwegian (2000–2020) and Barents Sea (2004–2020). We detected an overall significant northward shift of the marine fish community biomass in the North Sea, and individual species northward shifts in the Barents and North Seas, in 20% and 25% of the species’ biomass centroids in each respective region. We did not detect overall community shifts in the Norwegian Sea, where two species (8%) shifted in each direction (northwards and southwards). Among 9 biological traits, species biogeographic assignation, preferred temperature, age at maturity and maximum depth were significant explanatory variables for species latitudinal shifts in some of the study areas, and Arctic species shifted significantly faster than boreal species in the Barents Sea. Overall, our results suggest a strong influence of other factors, such as biological interactions, in determining several species’ recent geographic shifts.

## Introduction

Climate warming induces poleward expansions and equatorward range contractions of species’ geographic distributions on a global scale. These shifts have already been documented for thousands of species (Chaudhary et al., 2021; Pinsky, Selden & Kitchel, 2020). Marine species are expected to show stronger and faster niche shifts in response to global warming than terrestrial species because their geographic ranges more closely match their thermal limits, the marine environment is highly connected, and many marine species have huge dispersal potential as adults and/or planktonic life-stages (Pinsky et al., 2019; Sunday, Bates & Dulvy, 2012).

While the geographic distribution of over 50% of studied taxa shifts in response to climate warming, the direction and magnitude of these shifts depend on local abiotic variables, biological interactions and individual taxa characteristics (Pinsky et al., 2013; Tekwa, Watson & Pinsky, 2022). For each species, the probability of range expansion or contraction depends on several biological traits, including functional traits such as diet, swimming behaviour or habitat, but also biogeographic traits, such as distribution range size, or physiological traits, such as thermal preference or thermal range size (Kitchel et al., 2022; Perry et al., 2005; Sunday et al., 2015). Despite multiple studies, there is still little agreement on which specific traits facilitate or hinder climate-induced species distribution shifts. For example, fish species shifting distributions in the North Sea were detected to have faster life cycles and smaller body sizes than non-shifting species, while other studies showed that larger fish species with higher swimming ability are more prone to settle into temperate habitats from the tropics (Feary et al., 2014; Perry et al., 2005; Sunday et al., 2015). Contrasting trait-mediated response is also apparent at higher latitudes: while in the Bering Sea, no relationship was found between marine fish species dispersal-related traits and the degree of species thermal niche tracking, larger and generalist species expanded in the Barents Sea (Frainer et al., 2017; Fredston et al., 2021).

In the Arctic, a global warming hotspot, recent climate-warming-induced boreal species’ range expansions have affected fish species richness and food-web structure (Frainer et al., 2017, 2021; Gordó-Vilaseca et al., 2023; Kortsch et al., 2015). These included several species northward expansions in the Norwegian Sea, including the Atlantic mackerel (*Scomber scombrus*) and the European hake (*Merluccius merluccius*) (Skaret et al., 2015; Wienerroither et al., 2011), and at least 11 boreal species in the Barents Sea (Pecuchet et al., 2020). These community changes resulted in higher functional diversity and increased boreal-like trait profile in the Barents Sea (larger, longer lived and more piscivorous than Arctic species) (Frainer et al., 2017, 2021). However, changes in community traits towards an increased dominance of boreal-like traits in the Barents Sea do not necessarily indicate that these traits facilitate species distributional shifts in the area. The observed changes in the trait composition of the Barents Sea fish communities can be due to the overall community shifting, *i.e*., all species within the boreal community expanded poleward, or it can result from the range-shift of only few boreal species sharing shifting-related traits. Which of these processes dominate is unknown as the direct relationship between species traits and the magnitude of their climate-induced geographic shift has never been addressed in this region. Moreover, although previous studies confirmed the presence of species shifts (Fossheim et al., 2015; Pecuchet et al., 2020), a quantification of the magnitude and direction of these shifts is currently lacking. Understanding whether and which species respond faster to climate warming is crucial for informing effective conservation and management strategies (Pinsky, Selden & Kitchel, 2020).

**Figure 1:**
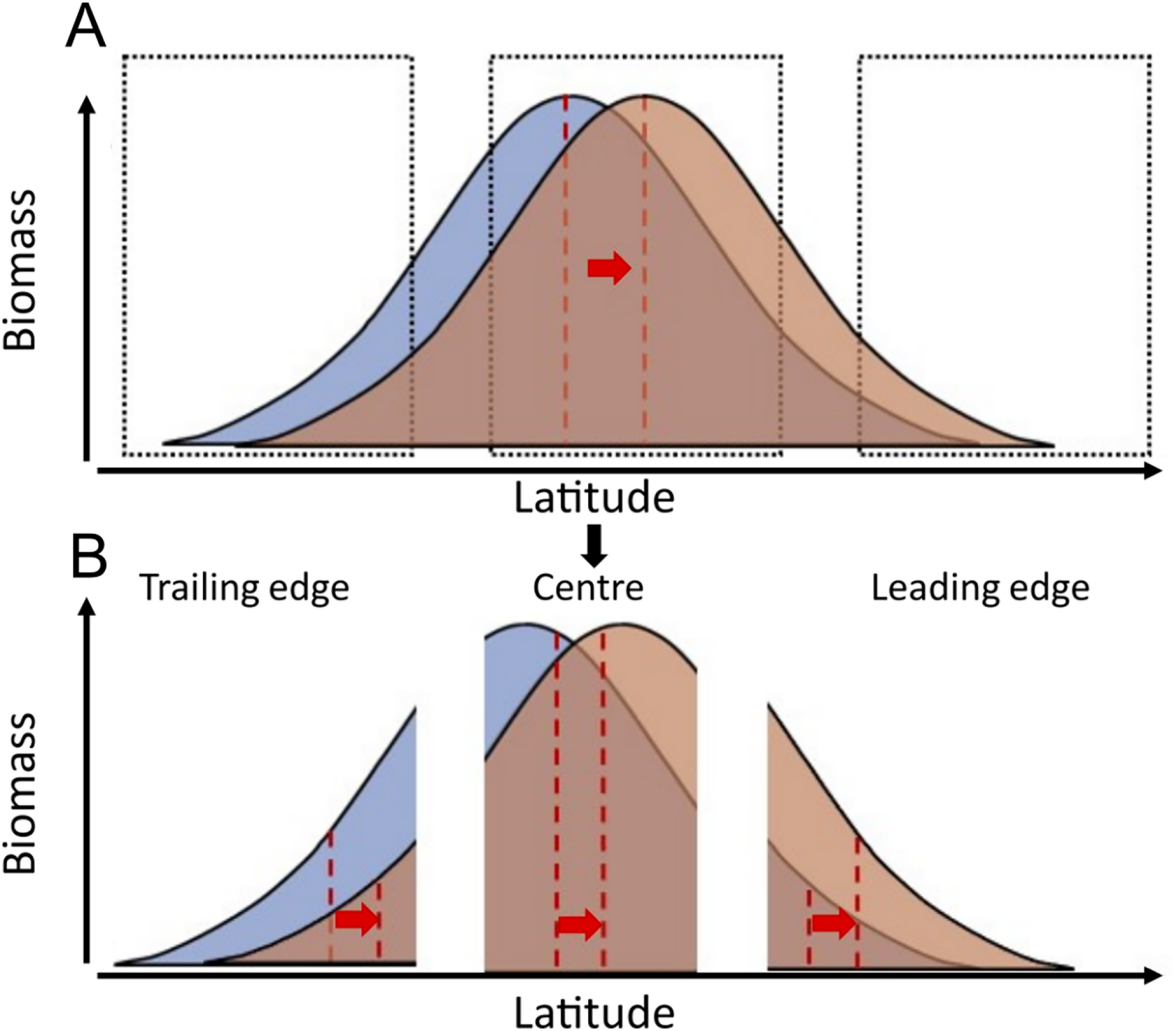
Conceptual representation of species distribution across a latitudinal gradient with warming (blue, before warming; red, after warming). Red dashed lines represent the biomass centroid before and after warming, calculated with the species’ full distributional range (A), or if they are calculated regionally at the trailing edge, the centre, or the leading edge of species distributions (B).

Species biomass is theoretically maximised at temperatures corresponding to the species’ optimal temperature and tend to decline at warmer and colder temperatures than this optimum (Burrows et al., 2019). According to this principle, assuming a linear relationship between temperature and latitude, and considering that temperature is the main driver of most species at biogeographic scales (Rutterford et al., 2023), the geographic distribution of species’ biomass centroids would be at latitudes where mean temperatures correspond to species’ “preferred temperatures”. As sea temperatures increase, these distributions may follow the increase in temperature across latitude. Areas that were previously too cold for a certain species, might approach species’ preferred temperatures, resulting in population increases. Conversely, areas that were previously at species’ preferred temperature might become too warm, resulting in population declines with warming in this region (Fig. 1).

Here, we analysed 9,522 research trawls to compare the pace of latitudinal range shifts across 83 marine fish species’ biomass centroids, from the North Sea (data from 1998 to 2020) the Norwegian Sea (data from 2000 to 2020), and the Barents Sea (data from 2004 to 2020). We explored the relationship between species’ geographic shifts, their ability to track thermal envelope shifts, and the relationship between species traits and their observed geographic shifts. To do so, we calculate (1) each species’ geographic latitudinal shift in km/yr, (2) each species’ thermal envelope latitudinal shift in km/yr, (3) the difference between these two as a measure of the tracking capacity of the species, and (4) we study to what extent can these geographic shifts be explained by species traits. We hypothesize that marine fish species will generally track their thermal envelope, as observed in other areas (Pinsky, Selden & Kitchel, 2020), and that species with higher dispersal potential (higher fecundity and pelagic habitat) and smaller body lengths show faster geographic shifts following shifts in their geographic thermal envelope, than large and slower maturing fish species, in accordance with previous studies in the North Sea (Perry et al., 2005). Although our data covers several species distribution ranges only partially, species range shifts should still be detected when occurring, if the relationship between species’ biomass and latitude follows a bell-shape curve, which is a wide-spread assumption (Burrows et al., 2019) (Fig. 1).

## Methods

### Study area

**Figure 2:**
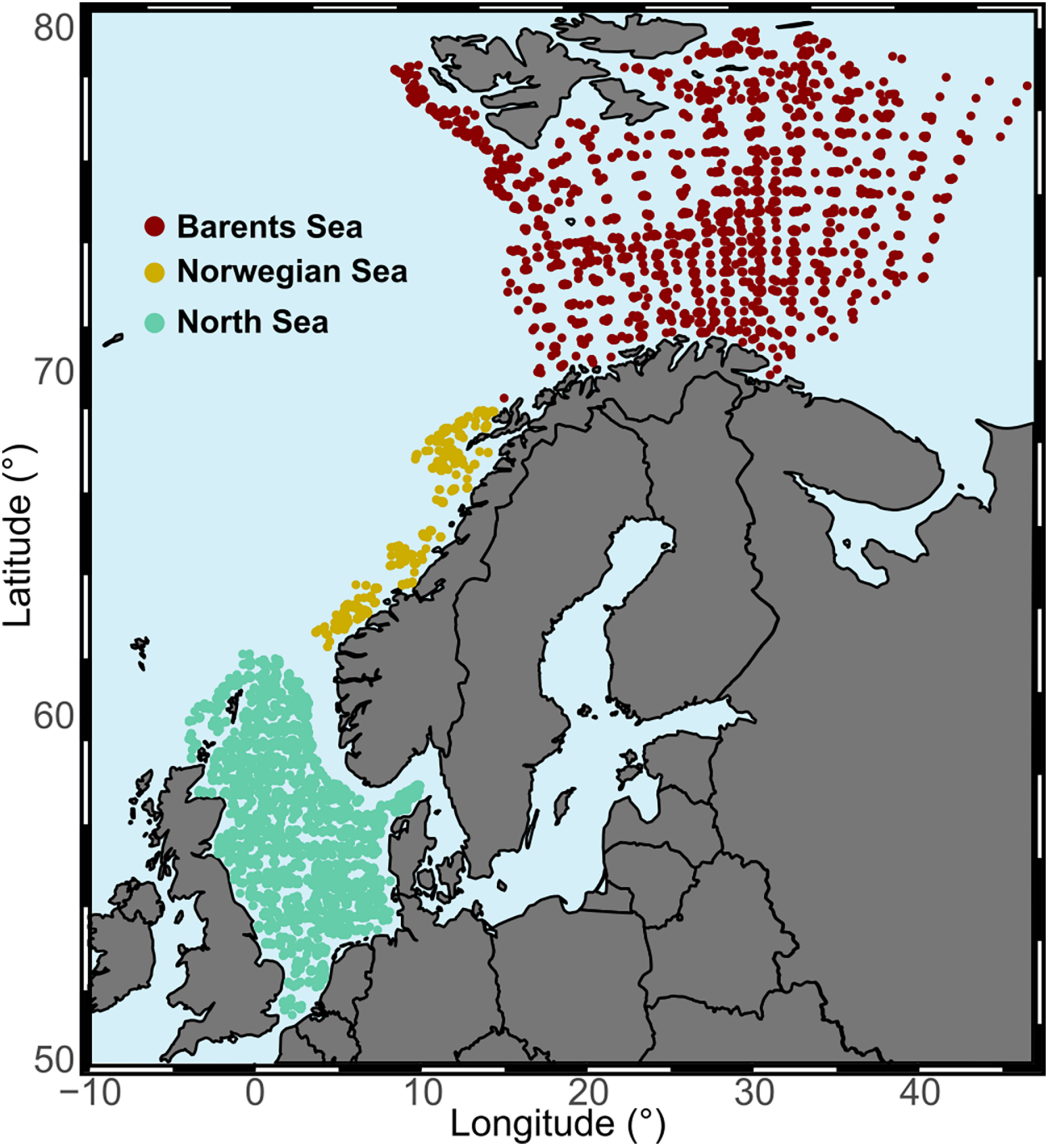
Study regions. Aggregated surveys at each study area across the full temporal range of the study.

Our study area was the continental shelf and slope from the North Sea into the Arctic Ocean, from 51°N to 80°N latitude, and from 4°W to 47°E longitude. The area comprises a temperature gradient, with average annual bottom water temperatures over 8 °C in the North Sea to −1 °C in the northern region of the Barents Sea. Based on different ecoregions (Costello et al., 2017; Yaragina & Dolgov, 2009), but also on temporal and spatial distribution of the data, we distinguished three study regions due to data collection differences between them (Fig. 2, Table 1 and Fig. S1). We restricted the analysis to offshore areas defined as being at least 20 km from the coast.

**Table 1:** Data included in each study area.

Region	First year	Last year	Years surveyed	Months surveyed	Hauls
Barents Sea	2004	2020	14 (not 2014 and 2016)	July, August and September	2,306
Norwegian Sea	2000	2020	21	October, November and December	628
North Sea	1998	2020	22	July, August and September	6,588

### Trawling data

We used two datasets in this study: The ICES DATRAS database for the North Sea between 1998 and 2020, accessed through the pre-treated FishGlob database (Maureaud et al., 2023), and the Norwegian bottom-trawl surveys database for the Norwegian and Barents Sea (IMR, 2021). Surveys were restricted from 2000 to 2020 in the Norwegian Sea, and from 2004 to 2020 in the Barents Sea. We visually inspected the data for a homogeneous spatial coverage of the study areas across time and discarded the years 2014 and 2016 in the Barents Sea, due to markedly different geographic distribution in those years. Surveys in the Barents Sea and North Sea were conducted during summer, while surveys in the Norwegian Sea were conducted in autumn (Table 1 and Fig. S1).

Both databases were standardised, and we excluded data associated with broken gear, had incomplete metadata (data without reporting depth, or coordinates, or type of gear), or were questionable (*e.g*., shrimp trawl opening of several kilometres). In the Norwegian and Barents Sea data, we restricted the analysis to shrimp trawling data using 20 mm mesh size, a maximum of 5 km trawling distance and 60 m of trawl opening, from 30 to 600 m depth. To ensure homogeneous species identification effort over time, we restricted the analysis to the fish species from the “allowed list of species in the Barents Sea Ecosystem survey” (Johannesen et al., 2021). This resulted in a dataset including 147 marine fish species (classes Actinopterygii, Elasmobranchii, Holocephali, Myxini and Petromyzonti). In the North Sea, the data from the ICES DATRAS database was collected using a GOV survey trawl, at a speed of 4 knots for 30 min, from 10 to 400 m depth, using 20 mm mesh size, and contained 129 marine fish species. For each species-region combinations, to be able to estimate the species mean latitude per year, we only kept the years where the species was recorded in at least five trawls in the region. Then, we excluded rare species by filtering out all species that had less than 10 individual years of data within each region. The final dataset contained 83 unique species across the three regions, with some species present in multiple regions, which resulted to a total of 119 unique species-region combinations with time-series between 15–22 years.

### Environmental data

We obtained environmental data for each region, and for each year we took the average of the three months of the season when each survey was conducted. Environmental layers were obtained from the Global Ocean Physics Reanalysis available through the Marine Copernicus repository (European Union-Copernicus Marine Service, 2018), and included sea surface temperature (SST), sea bottom temperature (SBT) and sea ice concentration (SIC) at a resolution of 0.083 degrees. Bottom depth was obtained from BioOracle at a resolution of 10 km. Environmental information was obtained for each sampling point coordinates using the function “extract” of the R package “raster” (Hijmans et al., 2015).

### Functional traits

We selected nine species’ biological traits that could be related to species expansion potential (Perry et al., 2005; Sunday et al., 2015) including five functional traits: (1) maximum length (cm), (2) age at maturity (years), (3) fecundity (number of eggs), (4) habitat (demersal or pelagic), and (5) trophic level; two thermal envelope related traits: (6) preferred temperature (°C), and (7) temperature range (°C); and one bathymetric trait: (8) maximum depth. These traits were obtained from FishBase (Froese & Pauly, 2023). We finally created a zoogeography trait (9) assigning a general biogeographic classification at each species, of the following categories: “Arctic”, “Arctic-Boreal”, “Boreal”, and “Deep-water”, as classified in Mecklenburg et al. (2018), or from FishBase when the species were not present in the former reference (*i.e*., not present in Arctic latitudes), adding the categories “subtropical” and “temperate” to the list of possible categories.

### Statistical analysis

#### Species geographic shifts

We calculated the centroid of each species’ annual distribution as their annual biomass-weighted average latitude and depth (Pinsky et al., 2013). We then fitted a generalized additive model (GAM) to the latitude of each species’ centroid, with a smooth effect of depth (to allow for the non-linear response of biomass with depth), a fixed effect of year, and a fixed effect of the annual mean and median of all surveys’ latitude. These non-temperature terms are used to partly correct for the spatio-temporal heterogeneity in the surveys.

The fixed effect of year in each of the models was considered as the latitudinal geographic shift rate, which was reported as km/yr, where each degree of latitude is converted to Km by multiplying by 111. The number of points used to calculate species’ centroids, and the number of years that a species was recorded, is summarised in Table S1, showing the 10 species with the highest and lowest amount of data (Table S1). We tested normality of shifting rates with Shapiro-Wilk test, and the differences of the whole community shifts from 0 with either parametric t-test, or Wilcoxon signed rank tests, depending on the fulfilling or not of the normality assumption (Shapiro & Wilk, 1965; Wilcoxon, 1945). We report the (pseudo) median and 95% CI of species’ latitudinal shifts because two out of the three areas did not fulfil the normality assumption.

#### Shift in species’ thermal envelopes

To study the ability of each species to track climate warming, we recorded spatial shifts in each species most favourable thermal conditions. To do so, we (1) estimated each species’ realized thermal niche, which we refer to as the species’ thermal envelope. Then, (2) spatially predicted biomass in each study area and year, (3) calculated the geographic centroid of this predictions every year, and (4) regressed the coordinates of these centroids with time, to obtain the annual thermal envelope shift for each species in the study area.

The thermal niche estimation was conducted using a commonly applied statistical approach, a two-part GAM model, to account for the large number of zeros in the data (83 × 2 models) (Pinsky et al., 2013; Wood, 2011). We assume that species realize thermal envelope, the relationship between species biomass and temperature, does not change during the study period, for which we estimate it using all the data for each species in one two-step model. Instead, what does change is the geographical distribution of this thermal envelope, which we estimate as each year spatial projection of this two-step GAM model, as we explain in the following lines.

Part one of the GAM was fit to presence/absence data with a binomial error distribution. Part two was fit to log(biomass) data for non-zero observations and had Gaussian errors (Pinsky et al., 2013). Explanatory factors in each model included bottom temperature, surface temperature, and depth, while average biomass for the year was included only in the biomass model. Penalized regression splines (maximum of 4 knots) were used for each term, as is typical in GAMs (Wood & Augustin, 2002). The depth was added to account for the effect of different surveyed depths across the dataset.

Thermal envelope predictions ( $b$) were then calculated as:

$b=pe^u\Phi$

where $p$ was the prediction from part one, $u$ was the prediction from part two, and was a correction factor, to correct for the re-transformation bias problem (Duan, 1983), and is calculated as:

$\Phi={\sum \nolimits_{i=1}^{n}e^{\varepsilon_i}\over{n}}$

where ${\varepsilon _i}$ were the $n\;$residuals from part two of the GAM for a given taxon (Duan, 1983; Pinsky et al., 2013).

For each model, we assessed its explanatory power using the AUC metric for the first step of the model (presence-only model), and the percentage of deviance explained in the second step of the model (biomass). Afterwards, for each study area, we spatially predicted species’ biomasses using environmental data from the season when each study area was surveyed, and the mean centroid of the predicted biomass was regressed against years to find each species’ thermal envelope shift rate in each study area. We assessed whether species’ geographic shifts tracked species’ thermal envelope shifts using a correlation test with Pearson correlation between the latitudinal shift in thermal envelope, and the species geographic latitudinal shift.

#### Species’ traits effect on geographic shifts, thermal envelope shifts and their difference

To study the influence of species’ traits on their geographical latitudinal shifts, we fitted generalised least square regressions with a power structure of the error to account for heteroscedasticity (GLS) to species geographic shifts. To test whether the number of each species’ number of records (rarity) affected the estimation of species’ latitudinal shifts, we regressed them with the number of records of each species as the only explanatory variable and included this variable in the GLS models with traits when it proved significant. Finally, we fitted generalised least square regressions to the species’ absolute latitudinal shifts, to test whether species’ number of records had a significant effect in the magnitude of these estimations, regardless of the direction. All analyses were conducted using the “nlme” package, and, for data wrangling, the “tidyverse” package (Pinheiro et al., 2022; Wickham et al., 2019).

### Results

**Figure 3:**
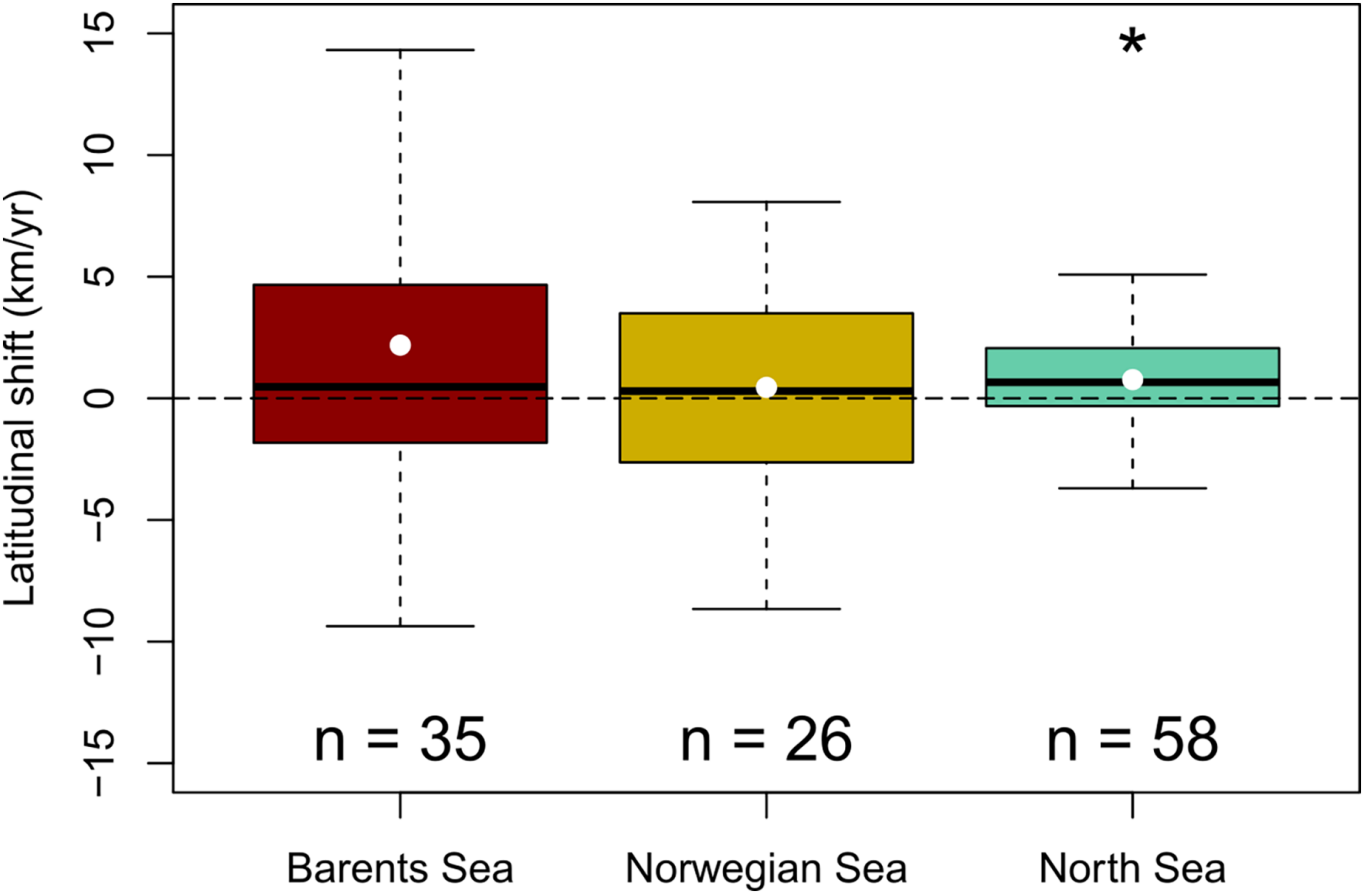
Geographic community shifts. Species individual geographic shifts at each study area (Barents Sea from 2004:2020, Norwegian Sea from 2000:2020, North Sea from 1994:2022). Asterisk (*) represents a mean and median shift significantly different from 0 (Student t-test in the Barents Sea, and Wilcoxon Rank test in the North Sea; *p*-val < 0.05).

A generalized northward shift of species’ biomass centroids was clearly detected over the last decades in the North Sea (from 1998 to 2022) (Wilcoxon signed rank test, *p* < 0.05), but not in the Norwegian Sea (from 2000 to 2020) (Student t-test, *p* = 0.6); nor in the Barents Sea (from 2004 to 2020) (Wilcoxon signed rank test, *p* = 0.09) (Fig. 3). The (pseudo) median shifting rates were 0.7 km/yr, 95% CI [0.1–1.4] in the North Sea, 0.4 km/yr, 95% CI [−1.3 to 2.2] in the Norwegian Sea, and 1.6 km/yr, 95% CI [−0.2 to 4.3] in the Barents Sea (Fig. 3). When each species was assessed individually, 15 species of 58 showed clear northward shifts of their biomass centroid in the North Sea (26%), while two species showed a clear southward shift (4%) (LR, *p* < 0.05) (Fig. S2). In the Norwegian Sea, two species were detected to shift northwards and two species southwards of a total of 26 species (8%) (LR, *p* < 0.05) (Fig. S3), and seven species of 35 showed clear northward shifts in the Barents Sea (20%), while two species showed a clear southward shift (6%) (LR, *p* < 0.05) (Fig. S4) (Table 2).

**Table 2:** Linear effect of Year on mean latitude of marine fish species in the Barents Sea (Bar), Norwegian Sea (Now) and North Sea (Nor).

Species	Reg.	*n*	Lat shift (km/yr)	*r* ^*2*^	*P*	Thermal envelope shift (km/yr)	*r* ^*2*^	*P*
*Amblyraja radiata*	Bar	15	3.49	0.68	0.25	0.05	0.64	0.00
*Anarhichas denticulatus*	Bar	15	−2.93	0.57	0.41	0.05	0.53	0.00
*Anarhichas lupus*	Bar	15	−0.51	0.13	0.91	0.03	0.29	0.00
*Anarhichas minor*	Bar	15	1.54	0.64	0.46	0.05	0.70	0.00
*Arctozenus risso*	Bar	14	−9.36	0.62	0.02	0.01	0.60	0.00
*Argentina silus*	Bar	15	3.81	0.52	0.08	0.02	0.42	0.00
*Artediellus atlanticus*	Bar	15	−2.55	0.84	0.19	0.02	0.42	0.00
*Boreogadus saida*	Bar	15	1.97	0.50	0.49	0.03	0.40	0.00
*Brosme brosme*	Bar	14	3.71	0.59	0.38	0.03	0.48	0.00
*Clupea harengus*	Bar	11	13.60	0.44	0.15	0.01	0.15	0.05
*Cottunculus microps*	Bar	15	5.52	0.50	0.15	−0.02	0.21	0.02
*Gadiculus argenteus*	Bar	13	1.79	0.67	0.27	0.02	0.54	0.00
*Gadus morhua*	Bar	15	−3.33	0.83	0.07	0.03	0.46	0.00
*Hippoglossoides platessoides*	Bar	15	−0.30	0.89	0.77	0.05	0.71	0.00
*Leptagonus decagonus*	Bar	15	5.72	0.91	0.00	0.04	0.65	0.00
*Leptoclinus maculatus*	Bar	15	0.17	0.58	0.92	0.03	0.55	0.00
*Liparis fabricii*	Bar	14	8.37	0.66	0.03	0.03	0.36	0.00
*Lumpenus lampretaeformis*	Bar	15	3.54	0.68	0.18	0.04	0.58	0.00
*Lycodes esmarkii*	Bar	10	1.17	0.86	0.82	0.05	0.59	0.00
*Lycodes gracilis*	Bar	15	−4.50	0.61	0.15	0.04	0.63	0.00
*Lycodes pallidus*	Bar	15	14.32	0.65	0.01	0.07	0.69	0.00
*Lycodes reticulatus*	Bar	15	11.90	0.67	0.03	0.03	0.49	0.00
*Lycodes rossi*	Bar	15	11.07	0.74	0.00	0.04	0.68	0.00
*Mallotus villosus*	Bar	15	0.12	0.65	0.96	0.05	0.64	0.00
*Melanogrammus aeglefinus*	Bar	15	−2.07	0.57	0.44	0.09	0.49	0.00
*Micromesistius poutassou*	Bar	15	−3.51	0.77	0.04	0.02	0.59	0.00
*Pollachius virens*	Bar	12	−1.58	0.71	0.39	0.02	0.55	0.00
*Rajella fyllae*	Bar	13	−4.68	0.74	0.11	0.01	0.15	0.05
*Reinhardtius hippoglossoides*	Bar	15	−0.47	0.72	0.87	0.00	0.03	0.42
*Sebastes mentella*	Bar	15	0.12	0.71	0.96	0.02	0.52	0.00
*Sebastes norvegicus*	Bar	15	−3.32	0.42	0.71	0.04	0.37	0.00
*Sebastes viviparus*	Bar	15	0.47	0.22	0.87	0.03	0.62	0.00
*Triglops murrayi*	Bar	15	12.23	0.61	0.03	0.03	0.74	0.00
*Triglops nybelini*	Bar	15	11.38	0.85	0.00	0.02	0.32	0.00
*Trisopterus esmarkii*	Bar	12	−0.32	0.25	0.93	0.02	0.59	0.00
*Anarhichas lupus*	Now	18	7.48	0.57	0.02	0.00	0.00	0.91
*Argentina silus*	Now	21	−0.73	0.76	0.52	−0.01	0.08	0.17
*Artediellus atlanticus*	Now	18	7.37	0.58	0.01	0.00	0.05	0.28
*Brosme brosme*	Now	20	4.29	0.67	0.07	0.00	0.10	0.12
*Chimaera monstrosa*	Now	21	−0.10	0.67	0.95	0.00	0.00	0.74
*Clupea harengus*	Now	12	−8.66	0.36	0.18	0.01	0.24	0.01
*Etmopterus spinax*	Now	17	−1.36	0.70	0.51	0.01	0.27	0.01
*Gadiculus argenteus*	Now	19	3.49	0.40	0.25	0.00	0.03	0.39
*Gadus morhua*	Now	21	−0.97	0.46	0.69	0.00	0.04	0.31
*Glyptocephalus cynoglossus*	Now	18	−3.26	0.36	0.38	0.00	0.01	0.67
*Hippoglossoides platessoides*	Now	20	0.07	0.45	0.98	−0.01	0.43	0.00
*Lepidorhombus whiffiagonis*	Now	17	8.07	0.53	0.09	0.04	0.74	0.00
*Lophius piscatorius*	Now	16	2.69	0.29	0.60	0.01	0.52	0.00
*Melanogrammus aeglefinus*	Now	21	−2.83	0.77	0.02	0.01	0.08	0.17
*Merlangius merlangus*	Now	20	1.56	0.53	0.58	0.02	0.44	0.00
*Merluccius merluccius*	Now	12	−5.63	0.59	0.41	0.03	0.75	0.00
*Micromesistius poutassou*	Now	20	−5.86	0.68	0.02	0.01	0.39	0.00
*Microstomus kitt*	Now	20	−2.63	0.73	0.20	0.00	0.08	0.17
*Molva molva*	Now	17	5.52	0.46	0.23	0.01	0.37	0.00
*Phycis blennoides*	Now	15	0.86	0.59	0.80	0.01	0.13	0.07
*Pollachius virens*	Now	21	−0.58	0.88	0.44	0.02	0.37	0.00
*Sebastes norvegicus*	Now	21	1.04	0.49	0.54	0.01	0.23	0.01
*Sebastes viviparus*	Now	21	1.47	0.75	0.19	0.01	0.23	0.01
*Triglops murrayi*	Now	17	4.13	0.41	0.37	0.02	0.21	0.02
*Trisopterus esmarkii*	Now	20	0.52	0.86	0.63	0.02	0.52	0.00
*Trisopterus minutus*	Now	15	−4.28	0.46	0.18	0.02	0.54	0.00
*Agonus cataphractus*	Nor	23	1.03	0.65	0.59	−0.01	0.17	0.04
*Amblyraja radiata*	Nor	23	−0.28	0.85	0.60	0.00	0.01	0.72
*Ammodytes marinus*	Nor	23	−4.22	0.82	0.01	0.00	0.09	0.15
*Anarhichas lupus*	Nor	22	2.41	0.63	0.04	0.00	0.04	0.31
*Arnoglossus laterna*	Nor	23	2.12	0.69	0.00	0.01	0.21	0.02
*Brosme brosme*	Nor	12	3.81	0.92	0.14	0.00	0.01	0.71
*Buglossidium luteum*	Nor	23	1.62	0.79	0.00	0.02	0.33	0.00
*Callionymus lyra*	Nor	23	0.67	0.82	0.26	0.00	0.19	0.02
*Chelidonichthys cuculus*	Nor	17	0.18	0.86	0.94	0.00	0.01	0.57
*Chelidonichthys lucerna*	Nor	23	−1.60	0.40	0.32	0.01	0.25	0.01
*Clupea harengus*	Nor	23	−0.16	0.83	0.66	0.01	0.24	0.01
*Echiichthys vipera*	Nor	23	0.82	0.46	0.21	0.01	0.05	0.27
*Enchelyopus cimbrius*	Nor	23	0.65	0.89	0.43	0.00	0.00	0.80
*Engraulis encrasicolus*	Nor	14	−0.80	0.74	0.71	0.01	0.05	0.27
*Eutrigla gurnardus*	Nor	23	0.43	0.90	0.20	0.01	0.13	0.07
*Gadiculus argenteus*	Nor	23	0.79	0.13	0.41	0.00	0.09	0.15
*Gadus morhua*	Nor	23	1.25	0.96	0.05	−0.01	0.11	0.09
*Glyptocephalus cynoglossus*	Nor	23	−0.02	0.73	0.98	−0.02	0.11	0.10
*Helicolenus dactylopterus*	Nor	14	9.06	0.65	0.06	−0.01	0.08	0.16
*Hippoglossoides platessoides*	Nor	23	0.54	0.93	0.06	0.00	0.00	0.94
*Hippoglossus hippoglossus*	Nor	13	7.97	0.78	0.04	0.00	0.01	0.55
*Hyperoplus lanceolatus*	Nor	23	0.09	0.38	0.90	−0.01	0.08	0.17
*Lepidorhombus whiffiagonis*	Nor	23	0.84	0.65	0.28	0.00	0.02	0.47
*Leucoraja naevus*	Nor	23	−1.62	0.60	0.16	0.01	0.06	0.22
*Limanda limanda*	Nor	23	−0.09	0.87	0.72	0.02	0.23	0.01
*Lophius piscatorius*	Nor	23	−0.84	0.93	0.24	−0.01	0.13	0.07
*Lumpenus lampretaeformis*	Nor	22	1.66	0.62	0.12	−0.04	0.22	0.01
*Maurolicus muelleri*	Nor	13	7.35	0.79	0.01	−0.03	0.15	0.05
*Melanogrammus aeglefinus*	Nor	23	0.97	0.93	0.01	0.00	0.01	0.59
*Merlangius merlangus*	Nor	23	−0.32	0.96	0.07	0.00	0.01	0.57
*Merluccius merluccius*	Nor	23	1.67	0.95	0.00	0.00	0.11	0.09
*Microchirus variegatus*	Nor	11	5.09	0.87	0.05	0.01	0.08	0.15
*Micromesistius poutassou*	Nor	23	2.02	0.58	0.13	−0.01	0.19	0.03
*Microstomus kitt*	Nor	23	0.16	0.87	0.55	0.00	0.06	0.22
*Molva molva*	Nor	23	2.06	0.78	0.02	−0.01	0.17	0.04
*Mullus surmuletus*	Nor	23	−0.29	0.42	0.88	0.01	0.04	0.31
*Myoxocephalus scorpius*	Nor	23	−2.37	0.62	0.18	0.00	0.00	0.73
*Myxine glutinosa*	Nor	23	3.40	0.56	0.01	0.00	0.03	0.39
*Platichthys flesus*	Nor	19	−3.70	0.58	0.09	−0.01	0.11	0.10
*Pleuronectes platessa*	Nor	23	−0.12	0.94	0.64	0.01	0.13	0.07
*Pollachius pollachius*	Nor	12	−4.50	0.66	0.30	−0.01	0.09	0.13
*Pollachius virens*	Nor	23	1.12	0.91	0.00	−0.01	0.14	0.06
*Raja clavata*	Nor	15	6.12	0.81	0.06	0.00	0.00	0.88
*Raja montagui*	Nor	16	5.07	0.77	0.10	0.00	0.00	0.94
*Scomber scombrus*	Nor	23	0.63	0.94	0.03	0.00	0.02	0.51
*Scophthalmus maximus*	Nor	23	−0.62	0.46	0.78	0.01	0.29	0.00
*Scophthalmus rhombus*	Nor	18	2.48	0.45	0.38	0.01	0.26	0.01
*Scyliorhinus canicula*	Nor	23	−1.64	0.80	0.11	0.01	0.06	0.21
*Sebastes viviparus*	Nor	20	0.71	0.71	0.74	0.00	0.00	0.74
*Solea solea*	Nor	23	5.84	0.49	0.05	0.01	0.29	0.00
*Sprattus sprattus*	Nor	23	−0.70	0.81	0.19	−0.01	0.09	0.14
*Squalus acanthias*	Nor	23	3.75	0.68	0.07	−0.01	0.03	0.44
*Trachinus draco*	Nor	13	−7.78	0.55	0.11	0.02	0.23	0.01
*Trachurus trachurus*	Nor	23	0.93	0.98	0.07	−0.04	0.13	0.07
*Trisopterus esmarkii*	Nor	23	0.20	0.72	0.69	−0.01	0.16	0.05
*Trisopterus luscus*	Nor	18	−3.17	0.57	0.03	0.01	0.04	0.33
*Trisopterus minutus*	Nor	23	4.22	0.97	0.00	0.00	0.01	0.70
*Zeus faber*	Nor	15	−10.27	0.82	0.10	0.02	0.13	0.07

**Note:**

Each model included a linear effect of year, overall annual mean and median latitude, and a smooth effect of depth, to correct for variability across surveys.

#### Thermal envelope models

Species thermal envelope models including SBT, SST, SIC and depth, presented a heterogeneous but reasonable fit to the data. Area Under the Curve (AUC) evaluation of taxa presence/absence model accuracy (part one of the GAM) ranged between 0.78 and 0.99 with a mean of 0.92. Models of non-zero observations (part two of the GAM) explained 1.9–54 % of the deviance in the data, with a mean of 21 % (Fig. S5). Most of the individual species’ thermal envelopes moved poleward in the Barents and Norwegian Seas (94% and 84% respectively), but less than half in the North Sea (43%) (Table 2).

**Figure 4:**
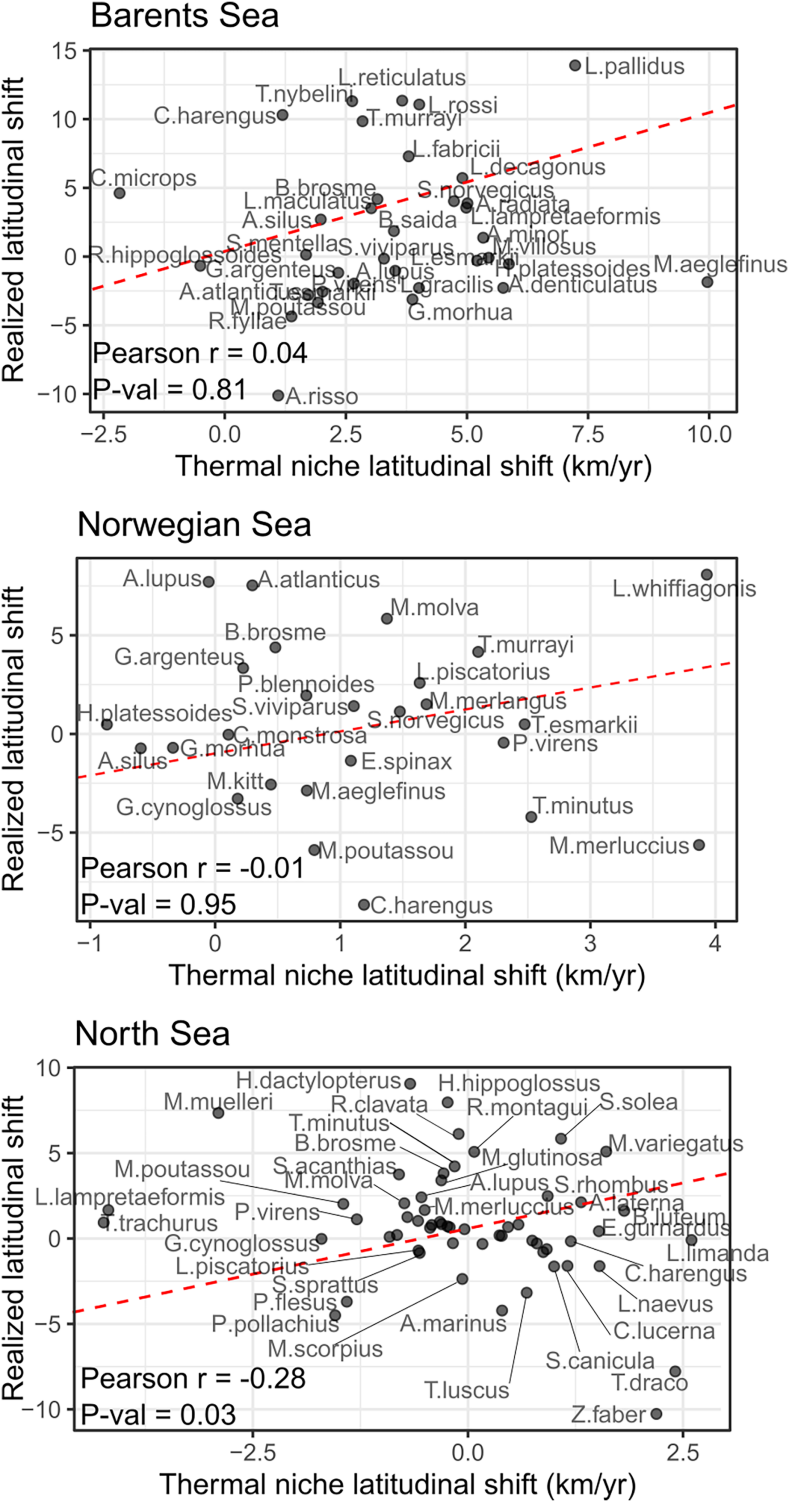
Correlation between species latitudinal geographic shifts, and species thermal envelope shifts from GAM model with bottom and surface temperatures. None of them showed significant correlation (Pearson correlation test, $\alpha$ = 0.05). Red line has slope = 1, and represents the line where species points would lay if there was a full correspondence between geographic shift and thermal envelope shift.

No significant correlation was detected between species shifting rates and their thermal envelope in the Norwegian and Barents Sea, and a negative significant correlation was detected in the North Sea (Pearson correlation = −0.28, *p*-val = 0.03) of the study areas (Fig. 4). In the Barents Sea, 60% of the species shifted in the same direction as their thermal envelope, and 95% of them did it northward (Table 3). In the Norwegian Sea, 54% of species shifted in accordance with their thermal envelope, 86% of them northward (Table 3). In the North Sea, 31% of the species shifted in the same direction as their thermal envelope, and of these, 61% shifted northward.

**Table 3:** Percentage of correspondence between latitudinal direction of observed change *vs* thermal envelope change in each study area.

Direction	Barents Sea	Norwegian Sea	North Sea
Observed shift ↓ but thermal envelope shift ↑	37%	39%	24%
Observed shift ↑, thermal envelope shift ↓	3%	8%	45%
Observed + thermal envelope shifts ↓	3%	8%	12%
Observed + thermal envelope shifts ↑	57%	46%	19%

We detected a significant effect of species’ records in the absolute difference between the geographic and the thermal shifts, implying that both estimations were more different in poorly sampled species than for common and well-sampled species (LRs, *p*-val < 0.05). To include this in the analysis, we conducted the correlation tests between species geographical shifts and species shifts in thermal envelope, adding one species at a time ordered by species frequency of occurrence (correlation test with the three most abundant species, then the four most abundant species, the five most abundant, the six, *etc*.), a significant correlation was only detected in the North Sea, when including all species lead to a negative correlation between species’ thermal envelope shift, and species’ realized latitudinal shift (Fig. S6).

#### Functional traits contribution to explaining species’ shifts

Each species’ number of records had a significant negative effect on species’ latitudinal shifts in the Barents Sea, for which we included this variable in all trait regressions in that study area (weighted generalised least squares model, *p* < 0.05, Table S2). In the other study areas, no significant effect of species records was detected on species latitudinal shifts (weighted generalised least squares model, *p* > 0.05, Table S2).

**Figure 5:**
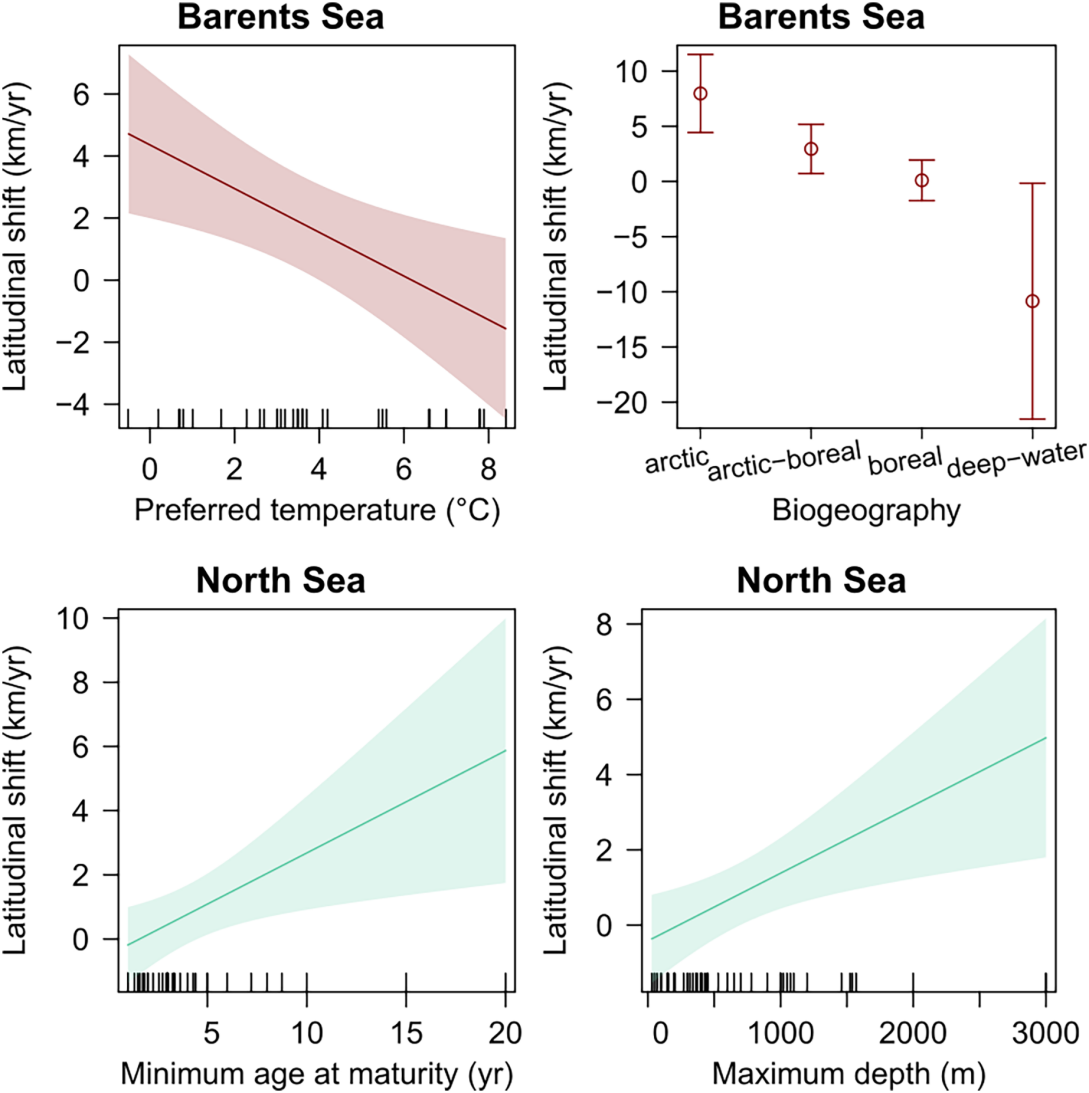
Species biological traits related to species latitudinal shifts in all study areas, which showed a significant effect in linear regression (*p*-val < 0.05).

Species’ biogeography, and preferred mean temperature were also significant explanatory variables for species latitudinal shifts in the Barents Sea, even when regressed in multiple regression including species records (weighted GLS, *p*-val < 0.05) (Table 4). Among these, species biogeography was a better explanatory variable in terms of explanatory power, and the only one significant when both were included in one multiple regression model, which suggest that Arctic species, and colder adapted species, shifted northward faster than warmer-water boreal species. In the North Sea, species’ minimum age at maturity and maximum depth were positively related to species latitudinal shifts, respectively (Table 4 and Fig. 5). Finally, no trait was detected to significantly explain species shifts in the Norwegian Sea (Table 4 and Fig. 5).

**Table 4:** Collection of traits that showed a signifcant (*p* < 0.05) and non-significant (showing only *p* < 0.05–0.1) relationship with species latitudinal shift in each region, using weighted generalised multiple least squared regression with each trait.

Study area	Trait	Slope		*r* ^*2*^	*p*
Barents Sea	Preferred temperature		−0.7 km yr^−1^ °C^−1^	0.25	<0.01
Barents Sea	Temperature range		−0.5 km yr^−1^ °C^−1^	0.20	0.1
Barents Sea	Habitat: pelagic (*vs* demersal)		−2.5 km yr^−1^	0.20	0.06
Barents Sea	Zoogeography: Arctic-Boreal (*vs* Arctic)		−5.0 km yr^−1^	0.58	0.02
Barents Sea	Zoogeography: Boreal (*vs* Arctic)		−7.9 km yr^−1^	0.58	<0.01
Barents Sea	Zoogeography: deep-water (*vs* Arctic)		−18.8 km yr^−1^	0.58	<0.01
Barents Sea	Trophic level		−3.1 km yr^−1^	0.28	0.07
Norwegian Sea	Preferred temperature		−0.8 km yr^−1^ °C^−1^	0.09	0.08
Norwegian Sea	Habitat: pelagic (*vs* demersal)		−2.8 km yr^−1^	0.09	0.1
North Sea	Minimum age at maturity		0.3 km yr^−1^ °C^-1^	0.12	0.01
North Sea	Maximum depth		0.002 km yr^−1^ m^−1^	0.10	<0.01

## Discussion and Conclusion

Our results show overall significant northward shifts in the North Seas but not in the Norwegian Sea nor in the Barents Sea, where community-wide species’ geographical shifts did not clearly differ from zero. However, the varying spatial footprint in these areas was higher than in the North Sea, and the sample size smaller (number of years of data, and number of species), which has an effect in the estimation of species geographical latitudinal shifts, and in the *p*-value calculation during hypothesis testing (Murtaugh, 2014). At the individual species level, for all areas we detected clear northward migrations for only a fraction of the species studied, when assessed at the species level. Even though there has been a borealization of the Barents Sea community in the last years (Fossheim et al., 2015; Gordó-Vilaseca et al., 2023; Pecuchet et al., 2020), obvious northward migration patterns for most species were not detected here, nor were they a decade ago (Aschan et al., 2013), which suggests that climate warming-induced species geographic shifts can be hard to detect at the individual species level but become apparent when considering enough species across a longer period. On the contrary, most species’ thermal envelopes shifted northwards in the Norwegian and Barents Sea, suggesting that either (1) species’ response to shifts in thermal envelope lag behind those shifts, or (2) the potential data gathering, and analysis artifacts challenge the detection of species’ realized geographical shifts more, than the detection of species’ thermal envelope shifts, or (3) a combination of those two processes is occurring.

Although several species shifted in the direction of their thermal envelope (most of which northward), no positive correlation between shifts and thermal envelope shifts was detected in any of the study areas, because several species whose thermal envelope shifted northwards, shifted in the opposite direction. For example, two pelagic species, blue whiting (*Micromesistius poutassou*) and spotted barracudina (*Arctozenus risso*), shifted southwards in the Barents Sea, even though their thermal envelope moved northwards. However, these species are pelagic, and this discrepancy could be due to the species changing their vertical distribution, which cannot be detected here using bottom-dwelling sampling gear. Overall, this lack of positive correlation, and even a negative correlation in the North Sea, between species’ shift in thermal envelope and species realized shift in distribution is both surprising, and partially line with previous global studies including the Barents Sea (Lenoir et al., 2020), and suggest a strong influence of other factors, such as biological interactions, in determining several species’ recent geographic shifts in the area. Moreover, we detected high variability in species shifting rates, showing that not all species in the community are responding coherently and homogeneously. Instead, some species are closely following their thermal envelope (*i.e*., polar cod (*Boreogadus saida*) in the Barents Sea, or whiting (*Merlangius merlangus*) in the Norwegian Sea), while others are shifting faster (*i.e*., Arctic eelpout (*Lycodes reticulatus*) in the Barents Sea) or slower (*i.e*., Pollock (*Pollachius pollachius*) in the North Sea) than would be expected by the geographic shift in their thermal envelope.

Some other studies have assessed the proportion and magnitude of species shifting within fish communities, and our results mostly align within them: In the Northeast United States continental shelf, 17 species of 36 studied (47%) shifted their biomass centroid poleward, six species of 29 studied (21%) also shifted northwards in the Californian Current System, 13 of 19 studied fish species in the Japan Sea (68%), and at least 20% of the whole fish community shifted poleward in south-eastern Australia, a climate-warming hotspot (Hsieh et al., 2008; Last et al., 2011; Nye et al., 2009; Tu, Tian & Hsieh, 2015). Lenoir et al. (2020) did not provide regional estimations of species shifts, but only a global estimation of approximately 5 km/yr of northward shifts in the global marine fauna, while Pinsky et al. (2013) estimated most of species northward or southward regional latitudinal shifts along the north American coast, to be of less than 2 km/yr (Lenoir et al., 2020; Pinsky et al., 2013). In Iceland’s sub-arctic coastal waters, 82 fish species showed distribution shifts at a mean northward rate of 1.6 km/yr during 22 years with warming conditions (Campana et al., 2020).

Within our study area, a previous study in the North Sea, described poleward species geographical shifts in 13 species (of 35 studied), mostly of <3 km/yr, between 1977 and 2001 (Perry et al., 2005). We detected significant poleward shifts in 15 species in the North Sea (of 58 studied), of similar magnitude (mostly <3 km/yr), from 1998 to 2020. Of the 13 species detected to shift northward in Perry et al. (2005), three of them continued to shift northwards in our study (*Arnoglossus laterna, Gadus morhua & Trisopterus minutus*), one showed a significant southward shift (*Trisopterus luscus*), and nine others did not show any trend (*Hippoglossoides platessoides, Merlangius merlangus, Eutrigla gurnardus, Lumpenus lampretaeformis, Lophius piscatorius, Enchelyopus cimbrius, Echiichthys vipera*, *Callionymus lyra & Limanda limanda*). Moreover, one of the two species detected to shift south in Perry et al. (2005), shifted northwards in our study (*Solea solea*), while the other was not detected to shift (*Trisopterus esmarkii*). Finally, from the 19 species showing no significant shifts in Perry et al. (2005), we detected seven significant northward shifts (*Anarhichus lupus*, *Buglossidium luteum*, *Melanogrammus aeglefinus, Merluccius merluccius, Molva molva, Myxine glutinosa, Pollachius virens*), while the remaining 12 species did not show distributional changes in our study either. Overall, even though we report similar absolute numbers of species shifting poleward, the relation to overall species included in the analyses differ substantially (25% of studied species shifts in our study, *vs* 36% in Perry et al. (2005)). Considering that both shared the same minimum data requirements (excluding species caught at less than five stations each year, during at least ten years), our results suggest that when a higher number of less-abundant species is included in the analysis, thanks to increased effort in sampling those species, and increased taxonomic effort, the absolute number of species shifting northwards is maintained, and the proportion of the community that shifts is therefore reduced. This estimation could further improve if enough data on the remaining species in the North Sea, which overall hosts over 220 species of marine fish, was available to be included in the analysis (Ducrotoy, Elliott & De Jonge, 2000).

In the Barents Sea, the only estimation of marine fish shifting rates previous to this study was published earlier this year, and its mean northward shift was estimated to be around 4.4 km/yr using Ecospace modelling and including 108 functional groups between 2004 and 2013 (Nascimento et al., 2023). We did not detect clear community-wide shifts in a smaller number of species during a longer time period (2004–2020), although the low *p*-value achieved when the whole community was considered deserves future reanalysis with more extensive data and species. The estimation of species shifts and shifting rates are often challenged by the low data availability of most species and study areas, which can result in wrong estimations of shifting rates because of the difficulty in discerning true range shifts from sampling variability artefacts (Bates et al., 2015; Brown et al., 2016). In the Barents Sea, the data availability remains very low for several species, despite the remarkably increasing sampling effort in the last decades. From the total pool of species, estimated to be of around 160 fish species (Mecklenburg et al., 2018), only about a fifth (35 species) met the data requirements to be included in this study, and 20% of them showed significant northward shifts. The differences between Nascimento et al. (2023) and our study may be due to different criteria in including/excluding rare species, as well as different study periods, and different methodological approach. Additionally, surveys in the Barents and Norwegian Seas are not spatially standardised, and different locations are sampled in several years (Johannesen et al., 2021). This introduces a source of bias that, although partly accounted for in our method (by explicitly accounting for different annual latitudes, and by filtering the raw data to keep the years with best spatial coverage), the effect of varying spatial footprint of the data cannot be completely eliminated.

Rare species are more prone to show false positive shifting rates due to fewer data points and higher variability in the biomass centroid estimation. This process occurs in both directions (poleward and equatorward) (Bates et al., 2015) and should not affect the central tendency of the estimated range shifts of a community. Even though we eliminated the rarest species, following common practice data curation criteria (Perry et al., 2005), this is likely the reason for which a significant negative effect of species number of records in species absolute shifting rate was detected in all study areas. However, in the Barents Sea, species number of records also showed a significant negative effect on non-absolute species shifting rates, which could indicate that rare species are shifting at faster rates than widespread species, maybe as a result of smaller niche breadth associated with higher vulnerability to climate-warming-induced habitat loss (Thuiller, Lavorel & Araújo, 2005). Increased vulnerability in rare Arctic species may induce a faster contraction of their trawling edge distribution, and therefore show a faster shift in the biomass centroid towards the north. However, higher uncertainty remains for species with less data where few records can have bigger influence on the centroid estimation, and further data collection focused on those species would help to confirm this hypothesis.

Regarding other species’ biological traits, several studies pointed to the lack of explanatory power of several live-history and biogeographic traits, for marine fish distribution shifts, including no effect of asymptotic length, age at maturation or geographical affinity in the Japan Sea, no effect of maximum length, length or age at maturation, fecundity or trophic level in the California Current System, and a very small contribution of size, trophic group or geographic range around Australian reef-fishes (Hsieh et al., 2008; Stuart-Smith et al., 2021; Tu, Tian & Hsieh, 2015). In line with these studies, we show that species’ life-history traits were mostly unable to capture species’ latitudinal shifts variability. However, species biogeography was key in the Barents Sea, with Arctic species shifting at a higher rate than boreal species, and with evidence of warmer-water species shifting faster than cold-water species. In the North Sea, we found that slower maturing species shifted faster than faster maturing species, which is the opposite of what previous studies showed did not detect any significant differences between maximum length or age at maturity between shifting and non-shifting species, despite previous results suggesting these differences (Perry et al., 2005). However, if only species significantly shifting northwards are considered, as done in Perry et al. (2005), no significant differences appeared in age at maturity between shifting and not shifting species. The inclusion of more species in the analysis, together with potential changes in the shifting community between study periods could explain these differences between studies.

The Barents Sea contains several different community assemblages separated across its thermal and bathymetric gradients which may respond differently to present and future warming and may show different vulnerability to species gains and losses (Husson et al., 2022; Johannesen et al., 2012; Wiedmann et al., 2014). Understanding how geographic shifts affect marine communities at a finer geographic scale within the study areas considered here remains a present and future challenge. Even though very recent studies report no clear sub-regional temporal community trends across the Barents Sea, these could be hard to detect due to interannual variability in community composition at smaller scales (Pecuchet et al., 2022). Moreover, our results contain surveys from only one season in each study region, which could be partially influenced by seasonal changes in species abundance and biomass, as observed in other marine ecosystems (Lloret-Lloret et al., 2021; Vilas et al., 2020). Future data collection at finer temporal and spatial scales would help to discern these community changes linked to geographic shifts from the natural (seasonal and spatial) variability of species communities.

## Supplemental Information

10.7717/peerj.15801/supp-1 Supplemental Information 1 Dataset for analysis.

10.7717/peerj.15801/supp-2 Supplemental Information 2 Supplementary tables and figures.

## Additional Information and Declarations

### Data Availability

The following information was supplied regarding data availability: Data and code are available at GitHub and Zenodo: https://github.com/CescGV/north_atlantic_shifts. CescGV. (2023). CescGV/north_atlantic_shifts: Code for “Over 20% of marine fishes shifting in the North and Barents Seas” (1.0.0). Zenodo. https://doi.org/10.5281/zenodo.8189002.
