# Supplementary material for: Over 20% of marine fishes shifting in the North and Barents Seas, but not in the Norwegian Sea
Source: PeerJ. 2023 Aug 31;11:e15801. doi: 10.7717/peerj.15801 (PMC10475276; doi:10.7717/peerj.15801)
Supplement: Supplemental Information 2 [file peerj-11-15801-s002.docx]

**Paper 2: Supplementary Material**

**
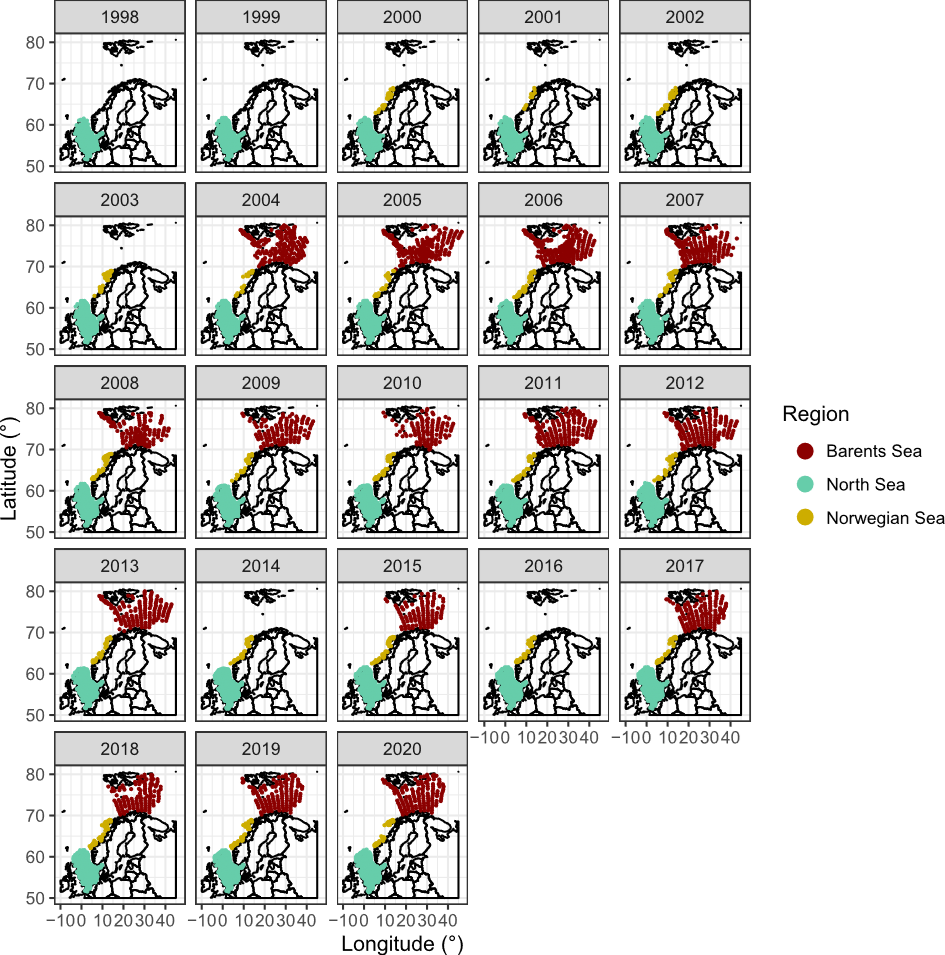
**

**Figure S1.** Spatiotemporal distribution of surveys in each region.


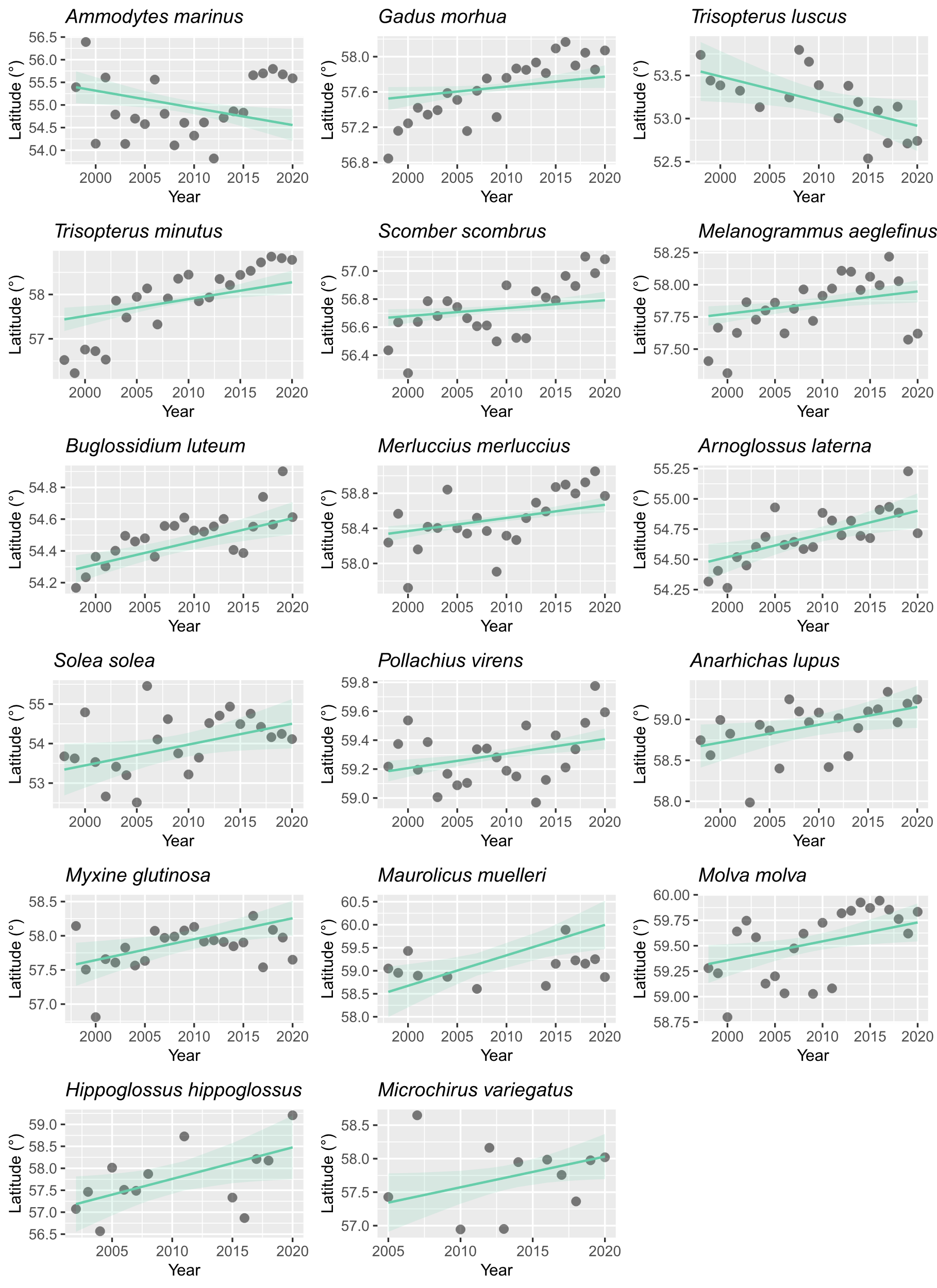


**Figure S2.** Significant shifts in species mean latitude across time in the North Sea


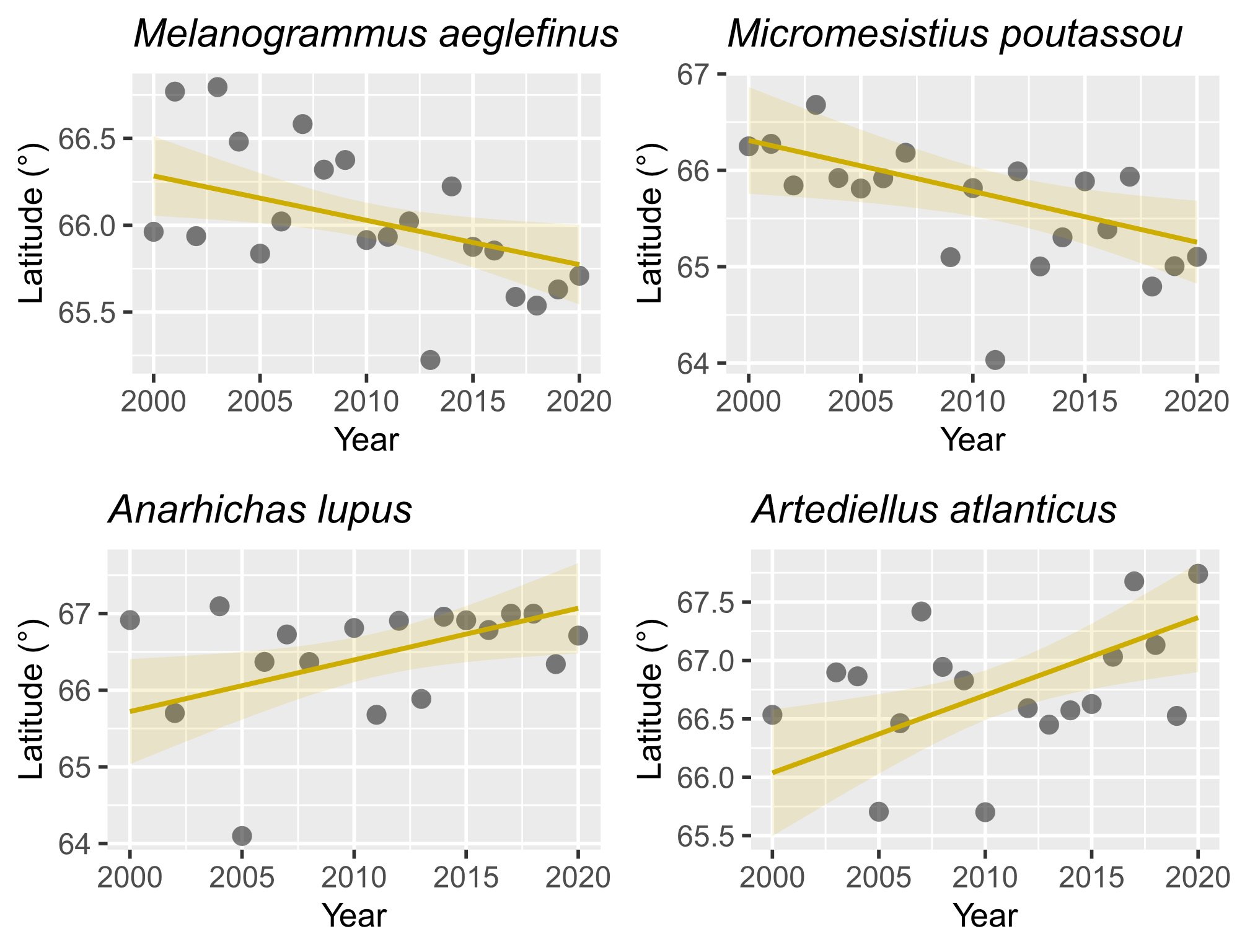


**Figure S3.** Significant shifts in species mean latitude across time in the Norwegian Sea


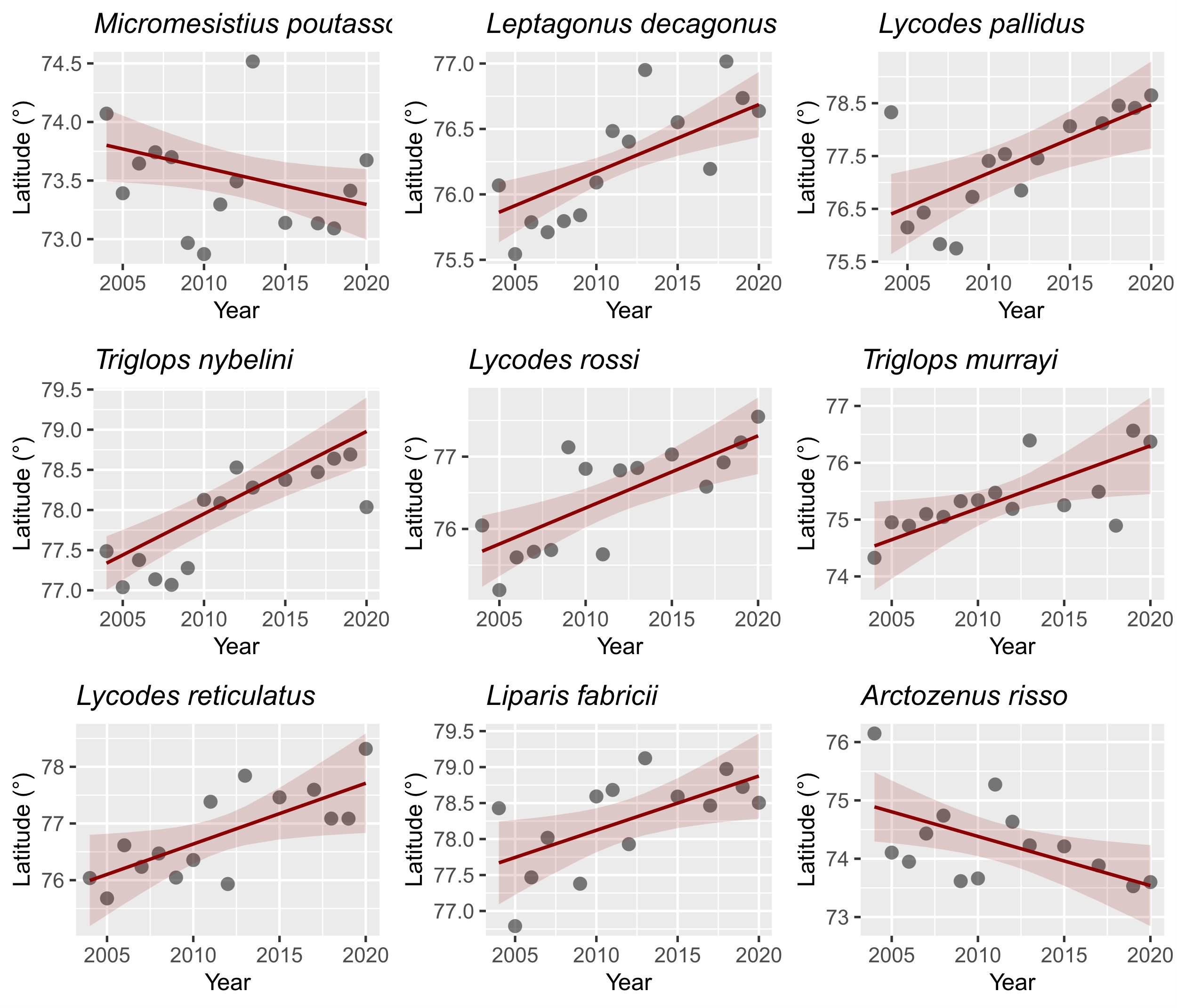


**Figure S4.** Significant shifts in species mean latitude across time in the Barents Sea

**
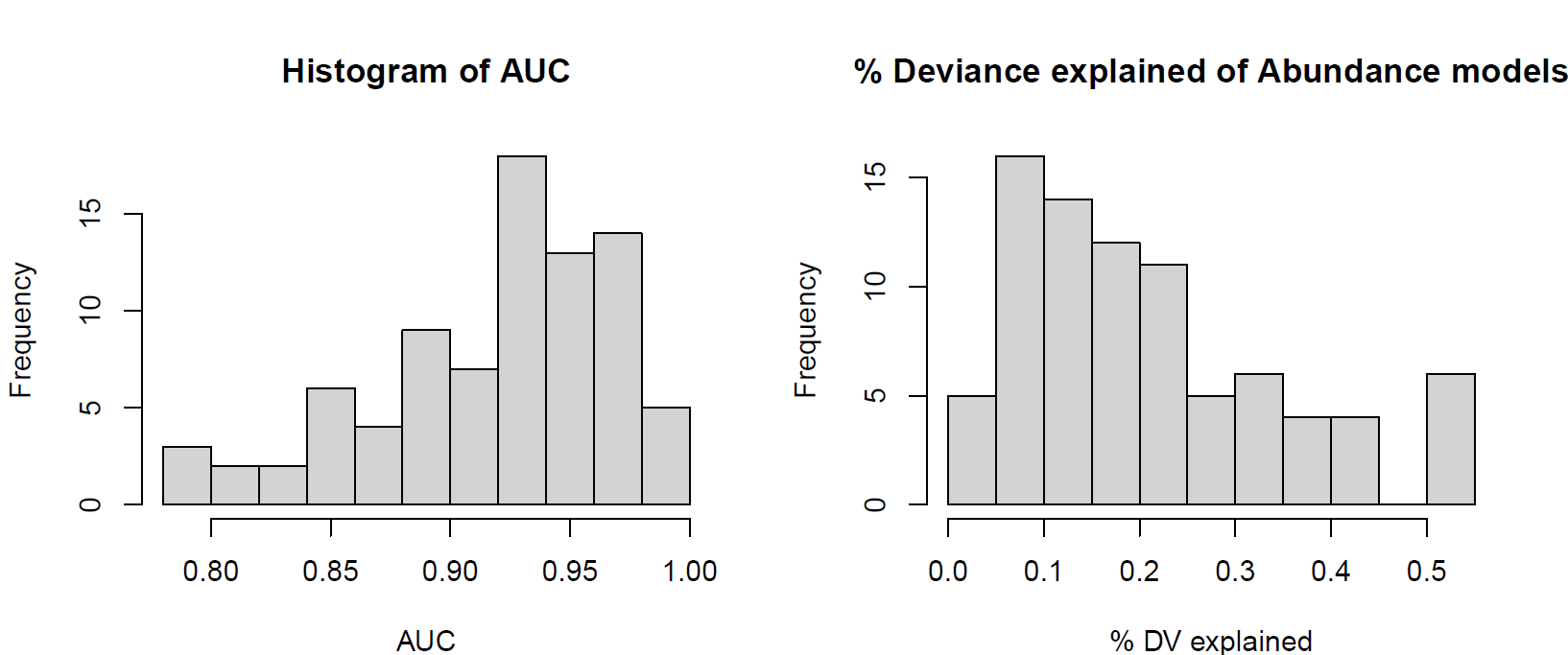
**

**Figure S5.** Histogram of all models AUC (step 1 of the two-step models, presence absence GAM binomial models) and %DV explained (Abundance log(Biomass) GAM models).

**
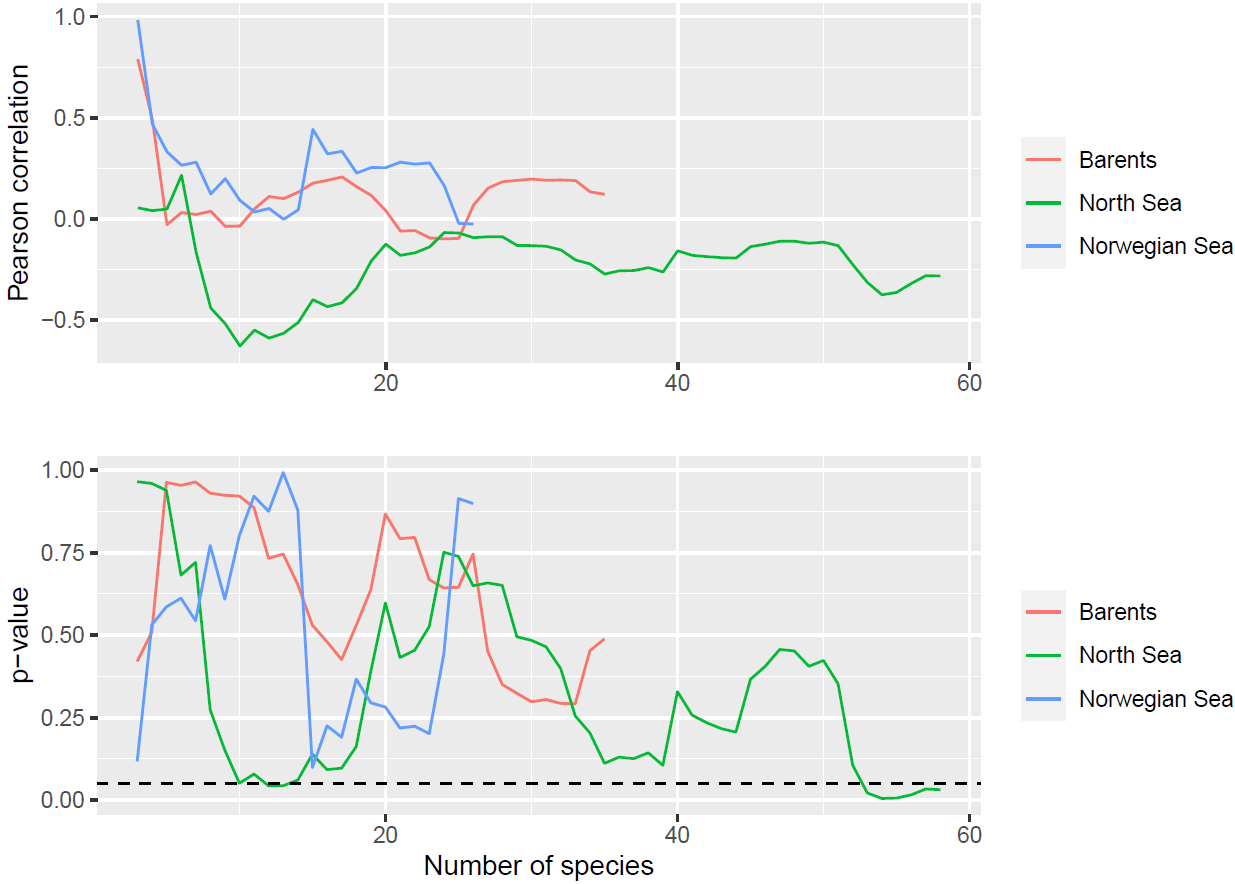
**

**Figure S6.** Correlation test results between species latitudinal geographic shifts and species latitudinal thermal envelope shifts at each study area, as the number of species included increases, from best sampled to worst sampled species.

**SUPPLEMENTARY TABLES**

**Table S1.** Number of sites from which each species geographic shifts and shifts in thermal envelope were calculated in the 10 least to most species in the surveys.

| **Index** | **Species** | **Region** | **Hauls** |
| --- | --- | --- | --- |
| 1 | *Brosme brosme* | North Sea | 73 |
| 2 | *Clupea harengus* | Norwegian Sea | 80 |
| 3 | *Microchirus variegatus* | North Sea | 83 |
| 4 | *Pollachius pollachius* | North Sea | 93 |
| 5 | *Merluccius merluccius* | Norwegian Sea | 98 |
| 6 | *Hippoglossus hippoglossus* | North Sea | 103 |
| 7 | *Lycodes esmarkii* | Barents Sea | 114 |
| 8 | *Trisopterus minutus* | Norwegian Sea | 118 |
| 9 | *Trachinus draco* | North Sea | 120 |
| 10 | *Lophius piscatorius* | Norwegian Sea | 122 |
| … | … | … | … |
| 110 | Gadus morhua | North Sea | 4113 |
| 111 | Scomber scombrus | North Sea | 4221 |
| 112 | Melanogrammus aeglefinus | North Sea | 4626 |
| 113 | Hippoglossoides platessoides | North Sea | 4664 |
| 114 | Eutrigla gurnardus | North Sea | 4815 |
| 115 | Pleuronectes platessa | North Sea | 4944 |
| 116 | Clupea harengus | North Sea | 5080 |
| 117 | Microstomus kitt | North Sea | 5097 |
| 118 | Limanda limanda | North Sea | 5512 |
| 119 | Merlangius merlangus | North Sea | 6229 |

**Table S2.**  Effect of species number of records in species latitudinal shift and absolute latitudinal shift in each region, using weighted generalised least squared regression.

| **Study area** | **Predicted variable** | **Estimate** | ***r^2^*** | ***P*** |
| --- | --- | --- | --- | --- |
| Barents Sea | Absolute shift rate | -3 x 10 -3 | 0.17 | **< 0.05** |
| Norwegian Sea | Absolute shift rate | -1 x 10 -2 | 0.36 | **< 0.05** |
| North Sea | Absolute shift rate | -7 x 10-4 | 0.26 | **< 0.05** |
| Barents Sea | Shift rate | -4 x 10 -3 | 0.10 | **< 0.05** |
| Norwegian Sea | Shift rate | 6 x 10 -3 | 0.001 | 0.9 |
| North Sea | Shift rate | 2 x 10 -4 | 0.006 | 0.5 |
